# A Robust Indoor Localization System Integrating Visual Localization Aided by CNN-Based Image Retrieval with Monte Carlo Localization

**DOI:** 10.3390/s19020249

**Published:** 2019-01-10

**Authors:** Song Xu, Wusheng Chou, Hongyi Dong

**Affiliations:** 1 School of Mechanical Engineering and Automation, Beihang University, Beijing 100191, China; wschou@buaa.edu.cn (W.C.); donghylucky@buaa.edu.cn (H.D.); 2 State Key Laboratory of Virtual Reality Technology and Systems, Beihang University, Beijing 100191, China

**Keywords:** indoor global localization, monocular camera, laser range finder, image retrieval, convolutional neural network, kidnapping

## Abstract

This paper proposes a novel multi-sensor-based indoor global localization system integrating visual localization aided by CNN-based image retrieval with a probabilistic localization approach. The global localization system consists of three parts: coarse place recognition, fine localization and re-localization from kidnapping. Coarse place recognition exploits a monocular camera to realize the initial localization based on image retrieval, in which off-the-shelf features extracted from a pre-trained Convolutional Neural Network (CNN) are adopted to determine the candidate locations of the robot. In the fine localization, a laser range finder is equipped to estimate the accurate pose of a mobile robot by means of an adaptive Monte Carlo localization, in which the candidate locations obtained by image retrieval are considered as seeds for initial random sampling. Additionally, to address the problem of robot kidnapping, we present a closed-loop localization mechanism to monitor the state of the robot in real time and make adaptive adjustments when the robot is kidnapped. The closed-loop mechanism effectively exploits the correlation of image sequences to realize the re-localization based on Long-Short Term Memory (LSTM) network. Extensive experiments were conducted and the results indicate that the proposed method not only exhibits great improvement on accuracy and speed, but also can recover from localization failures compared to two conventional localization methods.

## 1. Introduction

Global localization is a basic prerequisite for mobile robot navigation and control. The goal of mobile robot localization is to estimate the exact pose of mobile robot using only current sensor data based on a previously learned map. Accurate localization makes sense to many tasks such as motion control, path planning and target tracking [1,2,3].

In the past several years, extensive research has been conducted on indoor global localization, which could be divided into three categories from the perspective of the sensor: Wireless Local Area Network (WLAN)-based localization, laser-based localization and vision-based localization. WLAN-based localization realizes the localization process by combining the experience test and the signal propagation model based on the information of each network node, resulting in low cost but extremely susceptible to signal fluctuations and environmental interference [4,5]. In contrast, laser-based localization is more robust, in which the Bayesian filtering is leveraged to transform the mobile robot localization into the probability distribution estimation problem based on grid maps [6,7,8,9]. It not only achieves precise localization, but also enables continuous pose tracking of mobile robot. However, due to the presence of dynamic targets, frequent corrupted observations are prone to result in localization failures. With respect to vision-based localization, images can provide rich visual information such as geometric features and color textures [10,11,12,13,14], which is usually cast as image retrieval that finds the closest image in the geo-tagged database to the query image by means of feature matching. The retrieved geo-tagged image is likely to present the position where the query image is taken. Vision-based localization is widely applied to indoor localization due to its low cost, utmost convenience in use and rich information of geometric feature [15,16]. It is suitable for robot operation particularly in populated environments and does not suffer from the interferences often observed in light- or sound-based localization methods. However, it is hard to only use a camera for indoor localization because of the lack of distance information. Beside, its localization process is sensitive to the illumination and angle of objects.

The indoor environment tends to be highly similar in structure and layout. Images in different locations may contain the same object or repeated structural elements, resulting in global ambiguities, which is challenging for indoor localization if only through a single sensor. In this case, it is prone to cause mismatch if the localization is only performed by a single sensor. Therefore, it is necessary to fuse multi-sensor information for indoor localization [17,18,19].

In this paper, we propose a novel multi-sensor-based indoor global localization system. The proposed global localization system employs the coarse-to-fine mechanism to realize robust and efficient localization, which consists of coarse localization and fine localization. The coarse localization, which is cast as a multiclass place recognition problem, determines possible candidate locations where the robot may be located based on an image-based localization method, which comprises offline stage and online stage. In offline stage, we use a monocular camera to capture images by steering the mobile robot through the experiment environment, building a 2D geo-tagged image database applied for image retrieval. Each image in the database is labeled with ground truth pose information. Then, in online stage, the query image captured during the motion of robot is fed to the image retrieval system to determine the possible locations of robot. In the fine localization, a 2D laser range finder developed by HOKUYO in Japan is adopted to estimate the accurate pose of mobile robot by means of Monte Carlo localization based on the results of coarse localization. In addition, to address the “robot kidnapping” problem, a closed-loop mechanism is introduced to monitor the state of the robot in real time. When the mobile robot is kidnapped, the closed-loop mechanism will be activated rapidly and make adaptive adjustments to recognition the real location of robot based on the LSTM network with image sequences.

Our global localization system was tested and demonstrated in a real indoor environment. The experimental results show that the proposed system can perform better in different environments than the conventional localization method in terms of speed and accuracy for mobile robot. Besides, we further conducted robot-kidnapping experiments to verify that the proposed system provides the robot with the ability to recover from localization failure.

The remainder of this paper is organized as follows. Section 2 presents the related work. In Section 3, after describing the proposed indoor localization system, we elaborate the details of the coarse place recognition, fine localization and image sequence correlation with LSTM. Then, to verify the effectiveness and robustness of the proposed method, the results of various experiments are presented in Section 4. Finally, we conclude this paper in Section 5.

## 2. Related Work

### 2.1. Image Retrieval

Vision-based localization is usually cast as image retrieval that find the closest image in the geo-tagged database to the query image via feature matching [20,21,22,23,24]. Traditional image retrieval is mainly divided into text-based and content-based methods [25,26,27,28]. Text-based image retrieval [29,30] adopts text annotation to describe the content of images, which could be formed with keywords, such as objects, scenes, etc. When performing image retrieval, the system searches the images marked with the corresponding keywords according to the query keywords provided by the user. The text-based image retrieval method is only suitable for small-scale image data, requiring manual intervention in the labeling process. For precise queries, it is sometimes difficult for users to describe the images they want to obtain with short keywords. Besides, the manual annotation process is inevitably affected by the level of cognition, verbal use, and subjective judgment of the tagger, which will cause the difference in image description. Content-Based Image Retrieval (CBIR) was then proposed, establishing the image feature vector description based on low-level visual features of images such as color, shape and texture, which can be divided into global features and local features. The global features extracted from the visual content of the entire image are not suitable for scenes with similar layouts and are susceptible to the interference of occlusion and illumination. In contrast, the local features extracted from regions of interest exhibit greater discrimination and robustness. After more than a decade of development, many CBIR methods have been presented. The work in [31] proposes Hierarchical k-means for scalable recognition with a vocabulary tree. Zhu et al. [32] later adopted the Hierarchical k-means to build a large vocabulary tree for quantization and represent each video clip by a visual Bag-of-Words for instance search that outperformed all submissions on the instance search dataset TRECVID 2011. The authors believed that the use of larger vocabularies is the reason for better performance. In [33], Jegou et al. proposed Hamming embedding which could maintain the discriminative power of descriptor in the cluster. Then, Vector of Locally Aggregated Descriptors (VLAD) [34] was proposed by Jegou, which has good performance in large-scale image retrieval. However, the global descriptor extensively exploited in CBIR has difficulty achieving the desired performance in the case of illumination, deformation and occlusion, which compromise the retrieval accuracy.

Recently, CNN-based image retrieval [35,36,37,38] has gained popularity and gradually overtaken conventional image retrieval methods. CNNs trained with massive volume of data have been shown the superior ability to learn feature representations. In 2014, Babenko et al. [39] first proposed a CNN-based method for image retrieval based on fine-turning model. The work in [40] introduces a method to extract convolutional features from each layer of the networks, which are encoded into a vector of image features by VLAD. In [41], a regional maximum activation of convolution is built to aggregate several image regions into a compact feature vector, which is robust to scale and translation. Salvador et al. [42] extracted the features of images based on pre-trained Faster-RCNN and constructed the image descriptors via Image-wise pooling of activations and Region-wise pooling of activations for spatial re-ranking.

### 2.2. Monte Carlo Localization

Over the past few decades, extensive research has been conducted on the laser-based localization. blue Dellaert et al. [6] proposed a Monte Carlo Localization (MCL) with the particle filter algorithm based on laser rang scan that utilizes a discrete particle set to represent the posterior probability distribution, in which the correlation of the observation information is not considered, resulting in the degradation of the particle set. In response to the problem, many scholars have proposed different strategies for improvement. blue Merwe et al. [43] proposed to adopt the unscented Kalman filter to generate the particle filter importance density function, which does not need to calculate the Jacobian matrix, and the estimation accuracy is better. However, Unscented Kalman filter is susceptible to system noise and has poor adaptive characteristics. Kim et al. [44] proposed to update the Gaussian mixture model by incremental method and introduce a new weight calculation method to make the localization algorithm more robust. To reduce the computational burden, Zhang et al. [45] proposed a self-adaptive Monte Carlo localization for mobile robots by employing a pre-caching technique. The work in [9] proposes a global localization approach that merge SVM-based place recognition and particle filter for indoor environment with high similarity based on 2D range scan data.

In this paper, we adopt an adaptive Monte Carlo localization method to estimate the accurate pose of robot that dynamically adjusts the number of particles based on the observation information. It not only effectively avoids the particle degradation in global localization, but also prevents robots from being kidnapped.

## 3. Proposed Multi-Sensor-Based Indoor Localization System

**Figure 1:**
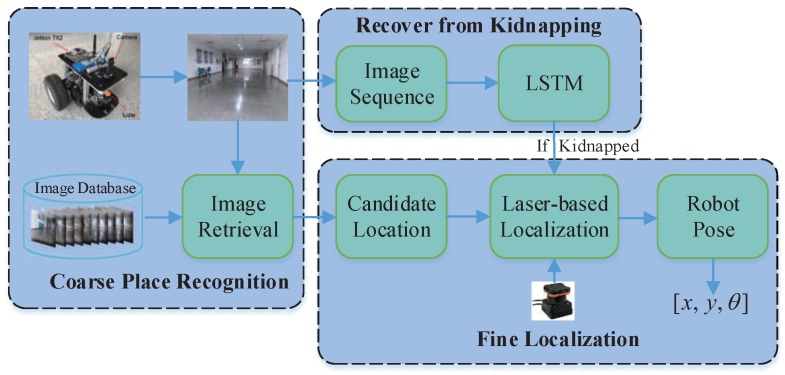
Overview of the proposed localization system.

The overview of the proposed localization system for mobile robot is shown in Figure 1. The global localization process consists of two stages: coarse place recognition and fine localization. In the coarse place recognition, the monocular camera is utilized to capture the image during the motion of the mobile robot and build a 2D geo-tagged image database applied for image retrieval. In the image retrieval, a pre-trained CNN for object detection is exploited to extract the high dimensional convolution features of images, which are adopted to search for images in the 2D geo-tagged image database most similar to the query image. The retrieved images are likely to present the possible locations of mobile robot, which are taken as the input to the next stage. In the fine localization, a 2D laser range is equipped to estimate the accurate pose of mobile robot by means of Monte Carlo localization, in which the candidate locations obtained from coarse place recognition are considered as seeds for initial random sampling. A set of sampled particles is injected into the candidate locations in a timely manner to track the real location of robot. In this process, the CNN-based image retrieval not only accelerates the convergence of samples in Monte Carlo localization, but also makes the global localization more robust in indoor environments with highly similar style and layout. Furthermore, to address the “robot kidnapping” problem, a closed-loop mechanism is introduced to monitor the state of the robot in real time. When the mobile robot is kidnapped, the closed-loop mechanism will be activated rapidly and make adaptive adjustments, which effectively exploits the correlation of image sequences via LSTM network. The LSTM network provides the localization system with a memory function ability to model sequence image data, which provides great discrimination for localization system in highly similar indoor environments.

### 3.1. Image-Based Localization

Image-based localization is usually cast as image retrieval process. There mainly exist two image-based localization methods: 2D image-based localization and 3D image-based localization [46]. The 2D image-based localization can be a component of 3D image-based localization to estimate where the query image might be taken. The 2D image-based localization searches the closest image in the geo-tagged database that presents the approximation position of mobile robot to the query image by means of feature matching. Then, the 3D image-based localization based on SFM is adopted to establish a set of 2D–3D matches to estimate the 6 DOF camera pose. However, building the 3D models required by structure-based techniques is not a trivial work, especially for large scale complex environment. Besides, the retrieval database image cannot provide valid information of local SFM model resulting in occasional localization failure. By contrast, 2D image-based localization methods only needs a geo-tagged images database, which is easy to construct.

In this paper, we adopt a 2D image-based localization methods to estimate the coarse location of mobile robot, which are effective in terms of computational efficiency and stability based on CNN-based image retrieval. As one of the transfer learning methods, fine-tuning based on the pre-trained model can transform the general features into special features, thus enabling the transferred features to adapt the tasks. To get better feature representation, an effective way is to explicitly learn weights suited for specific retrieval task based on pre-trained models. The work of Babenko [39] represents that models pre-trained on ImageNet for object classification could be improved by fine-tuning them on an external set of Landmarks images, even when using a classification loss. Inspired by Gordo [42], the pre-trained model of Faster R-CNN is used to extract the high dimensional convolution features of images, which searches the Top-N images with high similarity to the query image in the geo-tagged image database. The proposed image retrieval method can be divided into offline phase and online phase.

#### 3.1.1. Offline Phase

The dataset for image retrieval comprises 3550 images for training and 1032 images for testing with geo-referenced pose information, which is resized to 224 × 224 pixels. To generate ground truth pose information for each image, we steered the mobile robot through the indoor environment and captured the pictures from different positions. The positions of images were spaced roughly 2 m apart. At each position, we presented images at six different wide-angles covering full 360${}^{\circ }$. blue The dataset contains eight classes of common indoor objects, which can be used to construct local region descriptors for image retrieval.

During the training stage, we modified the output layers of the Faster RCNN to return nine class probabilities (eight classes in the dataset and an extra class for the background) and their corresponding regressed bounding box coordinates. Additionally, to obtain better feature representations for our image database, we chose to fine tune the model of Faster RCNN, which is considered as a feature extractor for image retrieval. In our experiments, we chose to update the weights of all layers after the first two convolutional layers to adapt to our query tasks. For the training details, we used a learning rate of 0.001 for 6000 iterations and 0.0001 for the next 2000 iterations. Weight decay and momentum were, respectively, set to 0.0005 and 0.9.

#### 3.1.2. Online Phase

**Figure 2:**
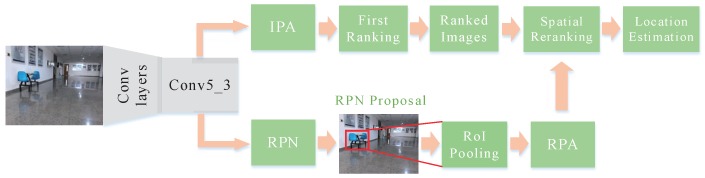
Architecture of the proposed image retrieval scheme.

Figure 2 shows the architecture of image retrieval. CNNs trained with massive volume of data have been demonstrated the superior ability to learn feature representations. During image retrieval, features extracted from different layers perform differently. According to Hao [47], the features extracted from top semantic layers are not conducive to object retrieval, which loses the object’s spatial information. In contrast, the activations of the convolutional layers may exhibit higher generalization ability and produce highly competitive compact image representations for object or scene retrieval. In fact, the fully connected layers can be removed in the process of exploiting intermediate layer activations as feature vectors and can remove the constraint of the input image size. In this paper, the conv5_3 layer of VGG16 is adopted to extract the features and to derive the representations for images. The extracted feature vector that represents the image to be localized after FC8 layer is one thousand dimensional. The process of image retrieval can be divided into two stages: initial filter stage and spatial re-ranking stage.

In the initial filter stage, Image-wise Pooling of Activations (IPA) strategy is used to construct global image descriptors for both query and database images, in which sum pooling is adopted for feature representation after the layer of feature extraction. Exploiting sum pooling to aggregate features from activations of the last convolutional layer has proven to have highly competitive performance [40]. In the test phase, we search for the Top-N images in the geo-tagged image database that are most similar to the query image based on the similarity measurement for first ranking. In this paper, we use the Euclidean distance to compute the distances between the descriptors of the query image and the descriptors of database images. In traditional image-based localization system, the candidate image with highest ranking in similarity is considered as the best match, and the position where the candidate image is located is returned to achieve the localization. However, for indoor environments, mismatching may occur in scenes with similar structures and layouts since the spatial relationship of feature vectors is neglected. Therefore, spatial re-ranking is necessary in image retrieval.

Post-processing with spatial verification has proven to be an effective way to improve the performance of image retrieval. After first ranking, the Top-N ranked images are verified for spatial re-ranking. In the re-ranking stage, Region-wise Pooling of Activations (RPA) with max pooling is adopted to construct the local region descriptors of images by aggregating the convolutional activations for each of the object proposals from the RoI pooling layer of Faster R-CNN. Then, we compare the local region descriptors of the query bounding box with the region-wise descriptors for all RPN proposals of the Top-N ranked images based on the Euclidean distance. For the high-level or complex scene, local feature representation exhibits superior generalization ability and strong distinction in image retrieval. For the convenience of comparison, we warp the bounding box to the size of the feature maps in the last convolutional layer. The region with maximum similarity for the Top-N ranked images provides the object localization and its score is kept for re-ranking. After the RoI pooling, the feature descriptors are l2-normalized and then whitening is applied to increase its robustness against noise.

### 3.2. Laser-Based Localization

In this section, we focus on the proposed fine localization method, which is based on an adaptive Monte Carlo localization. The adaptive Monte Carlo localization method approximates the posterior probability of the estimated state through a set of discrete weighted particles and gradually implements filtering through state prediction, update weight and resampling. The robot cannot recover from the localization failure when the pose suddenly changes. The traditional Monte Carlo localization method addresses this problem by adding random particles. Its localization accuracy is related to the number of particles. The more particles there are, the better the robustness is. However, the computational complexity caused by the increase of particles will affect the real-time performance of global localization. In this paper, our adaptive Monte Carlo localization method combines the augmented Monte Carlo method and the KLD-sampling, which not only solves the kidnapping problem, but also adopts KLD-sampling to dynamically adjust the particle number to reduce the computational complexity and improve localization efficiency. Its localization process can be performed as the following steps:

#### 3.2.1. Prediction Phase

In the prediction phase, a set of particles $S_{t}=\left\{\left(x_{\;t}^{\;(i)},\;\omega_{\;t}^{\;(i)}\right),\;i=1,2,\cdots ,N\right\}$ is randomly injected into all possible positions of the robot obtained by image-based localization method. The motion model $p\left(x_{t}^{}\left|x_{t-1},\;u_{t-1}\right.\right)$ is adopted to predict the current state of the robot in the form of a predictive Probability Density Function (PDF) $p\left(x_{t}^{}\left|z_{t}\right.\right)$ where $z_{t}$ represents the observation information at time *t*.

#### 3.2.2. Update Phase

In the update phase, the importance weight $\omega_{t}$ of each particle is obtained by the proposal distribution $\pi \left(x_{\;t}^{\left(i\right)}\left|m,x_{1:t-1}^{\left(i\right)},z_{1:t},u_{1:t-1}\right.\right)$, which is considered as the motion model $p\left(x_{t}\left|x_{t-1}\right.,u_{t-1}\right)$.

(1) $$\omega_{\;t}^{\left(i\right)}=\omega_{t-1}^{\left(i\right)}\frac{p\left(z_{t}{\left|\;x\right.}_{t}^{\left(i\right)},m\right)p\left(x_{t}^{\left(i\right)}\left|x_{t-1}^{\left(i\right)}\right.,u_{1:t-1}\right)}{\pi \left(x_{t}^{\left(i\right)}\left|m,x_{1:t-1}^{\left(i\right)},z_{1:t},u_{1:t-1}\right.\right)}=\omega_{t-1}^{\left(i\right)}p\left(z_{t}{\left|\;x\right.}_{t}^{\left(i\right)},m\right)$$

where $p\left(z_{t}{\left|\;x\right.}_{t}^{\left(i\right)},m\right)$ represents the observation model; *m* represents the a priori grid map; and $u_{t}-1$ denotes the control information at time t−1. The importance weights of each particle are updated using the observation model $p\left(z_{t}{\left|\;x\right.}_{t}^{\left(i\right)},m\right)$. In most cases, the sampled particles will gradually converge to a certain position. If the robot suddenly is kidnapped, the observation of the new particles is inaccurate. Additionally, the weight is normalized by the observation model and the difference is still obvious, which will lead to the localization failure. In this case, we inject random particles into the map when the observation of the particles is not very accurate based on augmented Monte Carlo method and assign the weight according to the observation model of the random particles. The quality of the current observation information is evaluated by comparing the long-term average weight of the particle set $\omega_{slow}$ with the short-term average weight of the particle set $\omega_{fast}$. They are calculated as follows

(2) $$\omega_{avg,\;t}=\frac{1}{N}\sum_{i=1}^{N}\omega_{t}^{\left(i\right)}$$

(3) $$\omega_{slow,\;t}=\alpha_{slow}\omega_{avg,\;t}+\left(1-\alpha_{slow}\right)\omega_{slow,\;t-1}$$

(4) $$\omega_{fast,\;t}=\alpha_{fast}\omega_{avg,\;t}+\left(1-\alpha_{fast}\right)\omega_{fast,\;t-1}$$

where $\omega_{a\;v\;g,\;t}$ is average weight of the particles at the current moment. Since the weight of sampled particles update is based on the observation model, the average weight can represent the quality of the current observation. The coefficients $\alpha_{slow}$ and $\alpha_{fast}$ are used to assign the ratio of the current average weight $\omega_{a\;v\;g,\;t}$, which is generally set to $0<\alpha_{slow}\leq \alpha_{fast}<1$. Then, the probability of adding particles during resampling is $\max\;\left\{0,\;1-\frac{\alpha_{fast}}{\alpha_{slow}}\right\}$. Therefore, when the long-term average weight is larger than the short-term average weight, the current average weight of the particles is small, the observation of the particle set is inaccurate and the probability of adding the random particles is higher.

After that, the particle set $\left\{x_{\;t}^{\left(i\right)},\omega_{\;t}^{\left(i\right)}\right\}$ is resampled. In the process of resampling, the weight of some particles is prone to be very small and almost can be ignored after several iterations, which is called particle degradation. Thus, many computations are wasted on particles that have little effect on estimating the state of sampled particles. Hence, we adopt KLD-sampling to solve the particle degradation. In the KLD-sampling, the Kullback–Leiber distance is used to measure the error between the true probability distribution and the approximation distribution. When uncertainty of the distribution is relatively high, that is, when the error between the true probability distribution and the approximation distribution is relatively large, we increase the number of sampled particles, which can ensure the robustness of the localization system, and vice versa. The KLD-sampling method dynamically adjusts the number of particles, ensuring that the distribution of current particles approximates the target distribution with a minimum number of particles. The minimum number of samples $N_{kld}$ is

(5) $$N_{kld}=\frac{N_{b}-1}{2\varepsilon }{\left\{1-\frac{2}{9\left(N_{b}-1\right)}+\sqrt{\frac{2}{9\left(N_{b}-1\right)}}z_{1-\delta }\right\}}^{3}$$

where $N_{b}$ denotes the number of subspaces occupied by sample; ε is the maximum value of the target distribution error; and 1−δ represents the probability that the error is less than ε. When the number of generated particles is larger than $N_{kld}$, the process of sampling can stop.

The process of the adaptive Monte Carlo localization is shown in Algorithm 1.

**Algorithm 1:** Adaptive Monte Carlo localization**Input:** observation information $z_{t}$, control information $u_{t}$;
**Output:**
$S_{t}$;
1:Initialization: $N_{r}=0$, $N_{m}=0$, $N_{kld}=0$, $N_{b}=0$, $\omega_{avg}=0$;2:**for** all b∈H **do** b=0;3:**end for**4:**if**$rand\left(\right)<max\left\{0,1-\omega_{fast}/\omega_{slow}\right\}$**then**5:    add random pose to $S_{t-1}$;6:    $N_{r}=N_{r}+1$;7:**else**8:    draw *i* with probability $\propto \omega_{t-1}^{\left(i\right)}$;9:    $N_{m}=N_{m}+1$;10:    $x_{t}^{\left(N_{m}\right)}$ = motion_model ($u_{t-1},x_{t}^{\left(i-1\right)}$);11:    $\omega_{t}^{\left(N_{m}\right)}$ = observation_model ($z_{t},x_{t}^{\left(N_{m}\right)},m$);12:    $S_{t}=S_{t}\cup x_{t}^{\left(i\right)},w_{t}^{\left(i\right)}$;13:    $\omega_{avg}=\omega_{avg}+\omega_{t}^{\left(i\right)}$;14:    **if** *b*($x_{t}^{\left(N_{m}\right)}$) = 0 **then**15:        *b*($x_{t}^{\left(N_{m}\right)}$) = 1;16:        $N_{b}=N_{b}+1$;17:        **if** $N_{b}>1$ **then**18:           $N_{kld}=\frac{N_{b}-1}{2\varepsilon }{\left\{1-\frac{2}{9\left(N_{b}-1\right)}+\sqrt{\frac{2}{9\left(N_{b}-1\right)}}z_{1-\delta }\right\}}^{3}$;19:        **end if**20:    **end if**21:**end if**22:**while** ($N_{m}<N_{kld}$ && $N_{r}+N_{m}<N_{max}$) or $N_{r}+N_{m}<N_{min}$ **do**23:    $\omega_{slow}$ = $\alpha_{slow}\omega_{avg}/N_{m}$ + ($1-\alpha_{slow}$)$\omega_{slow}$;24:    $\omega_{fast}$ = $\alpha_{fast}\omega_{avg}/N_{m}$ + ($1-\alpha_{fast}$)$\omega_{fast}$;25:**end while**26:**return**$S_{t}$;


### 3.3. Image Sequence Correlation with LSTM

LSTM is a recurrent neural network (RNN) with memory function that can process temporal sequence, allowing the network to choose to forget the previous hidden state or update the hidden state during the learning process. Compared with traditional RNN, gradient explosion and gradient disappearance can be effectively addressed in LSTM. It has been successfully applied to many tasks, such as action recognition [48] and language modeling [49] and translation [50]. Recently, integrating LSTM and CNN has become a common means of capturing long-term temporal dependencies in the computer vision community. The work in [51] applied spatial LSTM to human re-identification, analyzing the bounding box of pedestrian detection to learn the relevance of embedded feature space. LRCNs [52] present a novel end-to-end optimizable mapping from pixels to sentence-level natural language descriptions for video activity recognition by means of parsing the spatial and temporal dependencies.

**Figure 3:**
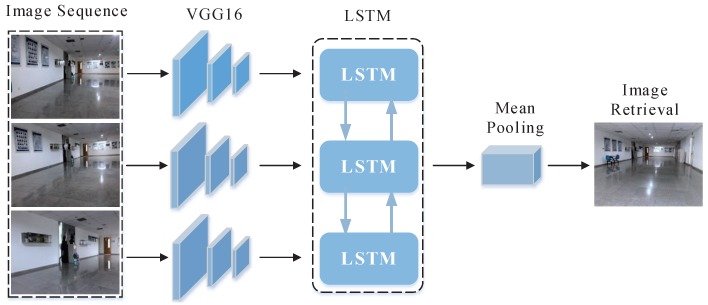
Architecture of the proposed LSTM network.

In this paper, LSTM and CNN are intelligently combined to better learn the contextual information of image sequences and to enhance the discriminative capability of the local features, which can effectively avoid mis-retrieval caused by insufficient feature information of single image. Due to the high similarity in structure and layout of the indoor environment, the robot are prone to be kidnapped, resulting in the failure of localization. To address the above problem, we embed the LSTM network in the global localization system, which is a parallel process to the previous coarse place recognition and fine localization. Once the robot is kidnapped, the LSTM network will be activated to restore the pose of robot via image sequence correlation. We determine whether the robot is kidnapped by measuring the probabilities of samples. If the maximum of probabilities of samples is less than a threshold, we assume that the robot has been kidnapped. The re-localization based on LSTM can determine the accurate position of the robot, which is then input into the adaptive Monte Carlo localization to recover and track the pose of robot. Its architecture is shown in Figure 3. After the robot is kidnapped, we use multiple frames of time continuous images captured during the motion of the robot to realize the re-localization. The pre-trained VGG16 is used to extract the feature of each query image. VGG16 has five convolution layers, five pooling layers and three fully connected layers. The extracted features vector that represents the image to be localized after FC8 layer is one thousand dimensional. The features of each frame are considered as one chunk for one input of LSTM. In this paper, the length of the sequence in LSTM is empirically set to six frames in order to achieve a balance between the accuracy and speed of localization. After the LSTM layer, a mean pooling is adopted to construct feature descriptors for feature representation. Since the dimensionality of the feature descriptor is relatively high, which is not conductive to direct comparison with the images in database, we apply PCA to reduce the dimensionality of the feature descriptors to improve retrieval efficiency and then whitening to enhance its robustness against noise. The Adam is adopted for optimization with NVIDIA Tian X. In the training stage, we use a learning rate of 0.001 for cost minimization and a batch size of 75.

## 4. Experiments

**Figure 4:**
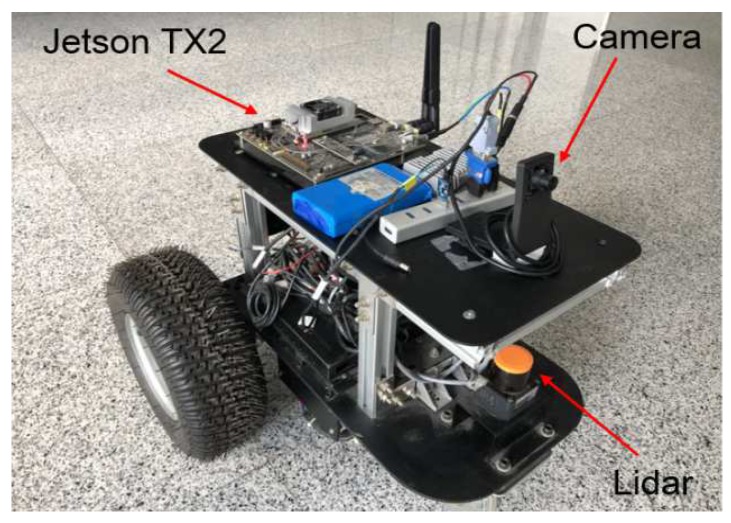
The mobile robot used to validate the proposed localization approach.

The above localization method was verified in the new main building of Beihang University and tested on a real mobile robot, as shown in Figure 4. The mobile robot is equipped with a monocular camera and a 2D laser range finder. The monocular camera used to capture the images in the front of the robot is 640 × 480 pixels in resolution. The laser developed by HOKUYO can cover 30 m and 270${}^{\circ }$. The localization method was processed on an Nvidia Jetson TX2 with 256 core-Pascal GPUs.

The experimental environment is about 80 m × 80 m in size. The testing dataset consists of 1032 images with 2D geo-tagged information blue (X,Y,θ) for each image. They were obtained by steering the mobile robot through the indoor environment and capturing pictures from different positions in the environment. The position of images are spaced roughly 2 m apart. At each position, we presented images at six different wide-angles covering 360${}^{\circ }$.

### 4.1. Experimental Setup

**Figure 5:**
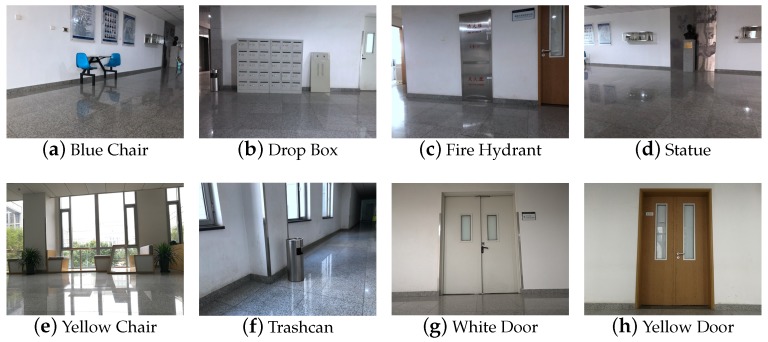
Classes for object detection in dataset.

To obtain the feature representations for image retrieval in coarse place recognition, we chose to fine-tune the architecture of Faster RCNN to output the regressed bounding box coordinates and the class scores for each geo-tagged image in the tested database. The following eight object categories were selected for object detection to construct local feature descriptors in image retrieval, as shown in Figure 5. The selected eight classes of objects are most common in indoor environments, facilitating image-based localization. Moreover, they contain rich and significant texture information, which is conductive to detection.

In the image-based localization, we searched the Top-50 images that are similar to the query image in the geo-tagged image database based on the Euclidean distance between global image descriptors constructed by IPA for first ranking. After first ranking, the ranked Top-50 images were verified for spatial re-ranking, in which RPA with max pooling was adopted to construct the local region descriptors of images. In the traditional image-based localization system, the candidate image with the highest ranking in the similarity is considered as the best match, and the position where the Top-N candidate image is located is returned to achieve the localization. However, for indoor environments, mismatching may occur in scenes with similar styles and layouts since the spatial relationship of feature vectors is neglected in image retrieval. In this experiment, the locations of the Top-4 candidate images of voting in order were all considered to be the possible locations of the mobile robot, and used as seeds for initial random sampling of Adaptive Monte Carlo localization. Then, the particles were randomly distributed at all possible initial location and the 2D fine pose of mobile robot was estimated based on probabilistic scan matching in conjunction with laser range scan and coarse pose information.

### 4.2. Coarse Place Recognition

**Figure 6:**
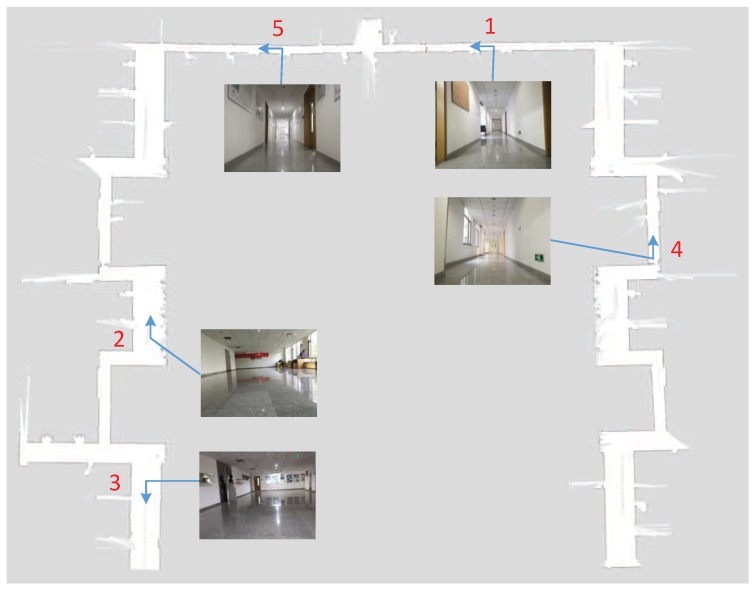
The grid map in the experimental site.

The experiment was carried out to evaluate the performance of image-based localization in detail. Figure 6 shows the grid map constructed by mobile robot on the third floor of the new main building of Beihang University. The experimental environment is mainly composed of indoor corridors, which are quasi-symmetrical, so positioning in such an environment is fairly challenging. There are many repetitive objects in the experimental environment such as chairs, doors and windows. The numerical size of the grid-cell is 5 cm × 5 cm. In this established grid map, the level of gray color denotes the occupancy probability. The darker is the color, the higher is the occupancy probability. The blue arrows represent the location and the direction of taking images in the indoor environment. Additionally, five images captured from different nodes are shown.

**Figure 7:**
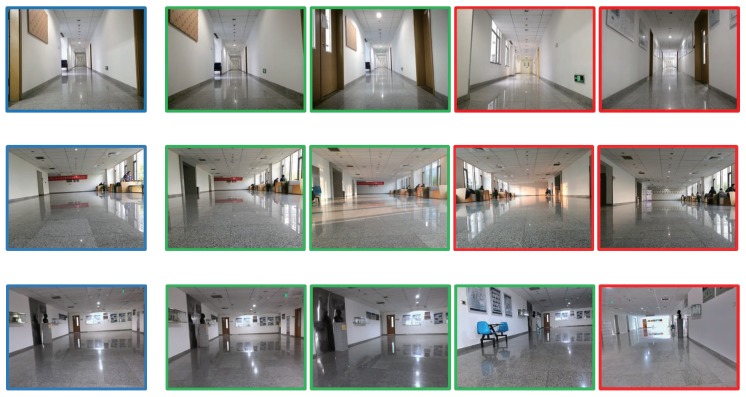
Three image retrieval results in the stage of coarse place recognition. The first column is the query image with a blue contour and the others are retrieved images. The images with red contour are mismatching.

Figure 7 presents some image retrieval results in the stage of coarse localization. For each row, the first image represents the query image taken by the mobile robot and the other four images are the re-ranked images. We adopted the image captured from node 1 as the query image for the localization experiment. At the testing stage, the MAP of the image retrieval method was about 0.742 for the Top-4 re-ranked images. For the query image in the first row of Figure 7, which corresponds to the image of node 1 in Figure 6, its Top-4 retrieved images correspond to four locations in the experimental environment. The first two candidate images located near node 1 are fairly accurate retrieval results, but the third and fourth candidate image taken from nodes 4 and 5 (in Figure 6) are mis-retrievals. Due to the structural similarity of the indoor environment and the interference of illumination and viewpoint, the occurrence of mis-retrieval is inevitable. In principle, the higher is the rank of the candidate image, the more similar it is to the query image. Therefore, the importance of the Top-4 re-ranked images is different. We defined the importance weight of the four retrieval images correspond to four candidate locations as 4:3:2:1.

### 4.3. Accurate Localization

#### 4.3.1. Accurate Localization Estimation

Based on the importance weight obtained by the coarse place recognition, a set of particles was randomly injected around these locations and output to the initialization process of Adaptive Monte Carlo localization.

We focused on verifying the accuracy of the localization system to estimate the pose of the mobile robot. In Table 1, the 2D pose (X,Y,θ) of node 1, node 4 and node 5 estimated by coarse place recognition was selected as the candidate pose for random sampling. After several iterations, most particles tend to converge to the real position of the robot and estimate the fine pose of robot. As shown in Table 1, candidate node 1 was determined as the correct node. Node 4 and node 5 were candidate nodes similar to node 1 in structure and layout. The fine pose of the robot estimated by the proposed localization method was $\left(X,Y,\theta \right)=\left(1572.23\;\mathrm{cm},\;3723.51\;\mathrm{cm},\;-179.64^{\circ }\right)$, and the ground truth pose of the robot was $\left(X,Y,\theta \right)=\left(1561.45\;\mathrm{cm},\;3730.72\;\mathrm{cm},\;-173.42^{\circ }\right)$ . It can be seen that the system could accurately estimate the pose of the robot and reliably keep track of it afterwards.

#### 4.3.2. Impact of the Image-Based Localization

**Figure 8:**
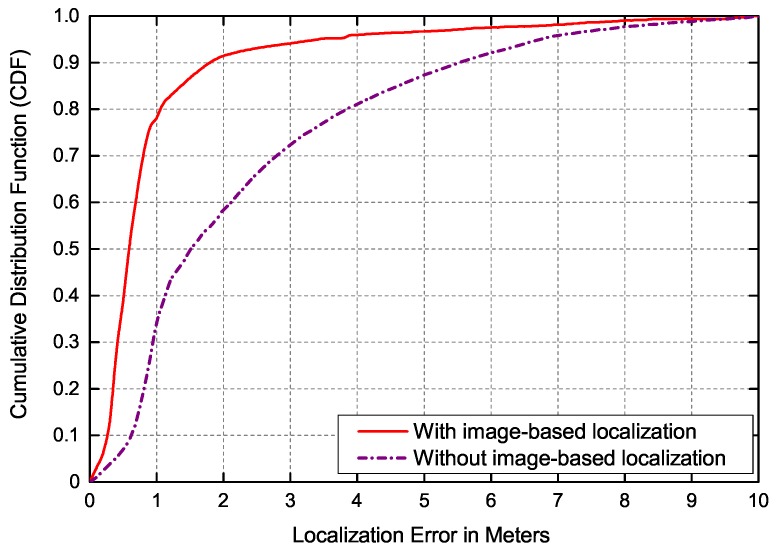
CDF of localization accuracy with and without image-based localization.

The coarse place recognition results obtained by image retrieval can provide the candidate locations of robot for Monte Carlo localization, which not only speeds up the convergence of sampled particles but also significantly improves the robustness of the localization system, especially in complex environments with high similarities. All sampled particles are randomly generated in arbitrary areas of the grid map with the same weight when the image-based localization is removed. To evaluate the contribution of the image retrieval system, the Cumulative Distribution Function (CDF) of the localization error with and without image-based localization is shown in Figure 8. The case of not performing the image-based localization reflects running Adaptive Monte Carlo localization without any prior information. As shown in Figure 8, the 90% localization error of the proposed method without image-based localization is less than 6 m. In contrast, the proposed localization with image-based localization can realize the localization process effectively. When the image retrieval is performed, the localization accuracy is fairly high and the 90% localization error is less than 2 m.

#### 4.3.3. Impact of the Laser-Based Localization

**Figure 9:**
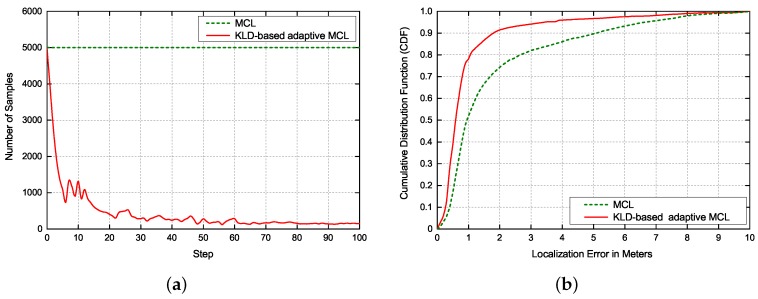
(**a**) Typical evolution of number of samples of two methods in global localization. (**b**) CDF of localization accuracy of two methods in global localization.

To verify the performance of the laser-based localization proposed in this paper, comparative experiments among Monte Carlo Localization (MCL) and KLD-based adaptive MCL were performed, as shown in Figure 9. After determining the coarse location of the robot obtained by image-based localization, we adopted MCL and KLD-based adaptive MCL for fine localization to estimate the accurate pose of robot. The initial number of samples in global localization was uniformly set to 5000. Figure 9a presents the typical evolution of the number of samples of the two methods in global localization. As can be seen, the MCL method utilizes fixed number of samples for importance sampling during the entire state estimate process while the KLD-based adaptive MCL dynamically adjusts the number of samples according to the underlying state uncertainty, which can significantly improve the efficiency and reduce the computational complexity. As expected, with the improvement of localization accuracy, the samples decrease gradually in the KLD-based adaptive MCL. Figure 9b shows the CDF of localization accuracy of two methods in global localization. Obviously, the performance of KLD-based adaptive MCL outperforms MCL in global localization accuracy. The 50% localization error of KLD-based adaptive MCL is less than 0.6 m, while 50% localization error of MCL is less than 0.95 m.

#### 4.3.4. Evaluation of Proposed Localization System

**Figure 10:**
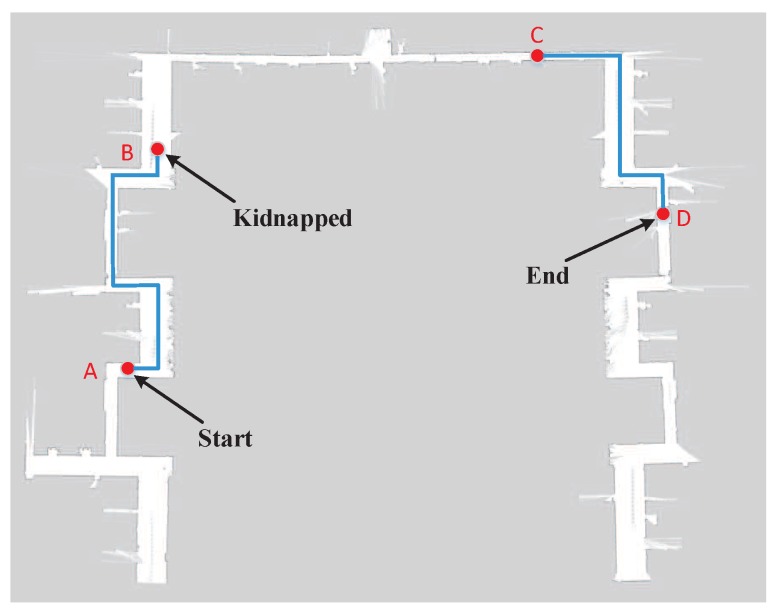
Localization error of three methods in global localization.

To quantitatively evaluate the performance of the proposed global localization method, we chose mixture Monte Carlo localization (MCL) [3], image-based localization [20] and the proposed global localization method for comparative experiments. The maximum number of particles in the Monte Carlo localization was uniformly set to 5000. In the process of Monte Carlo localization, the mean of the sampled particles was used to estimate the pose of robot. The localization error versus execution step of three localization methods is depicted in Figure 10. As can be seen, the proposed localization method could estimate the pose of the robot efficiently. Moreover, in the initial stage localization, compared with the other two localization methods, the localization error of the proposed method converges significantly more quickly, because the coarse localization results of image retrieval provides reliable initial pose information robot for the next accurate localization stage, which can accelerate the convergence of particles. In addition, the proposed method is overall more robust in localization process compared with the other two localization methods. The adaptive Monte Carlo localization method in this paper adjusts the distribution of particles according to the observation model of system. Specifically, the average localization error of the proposed method is less than 40 cm, which is enough to meet the requirements for indoor localization.

### 4.4. Re-Localization from Kidnapping

We focused on the ability of proposed localization system to deal with robot kidnapping which is a fairly tricky problem. To provide the robot with the ability to escape from kidnapping, we integrated the LSTM unit into CNN, so that the network can learn the feature correlation between image sequences. The embedding of the LSTM network will slow down the test speed. To reduce the computational cost of training and testing, we empirically limited the length of the sequence to six frames in this experiment, which not only ensures the recognition speed of the network, but also allows the captured image sequence to fall into the same area as much as possible.

**Figure 11:**
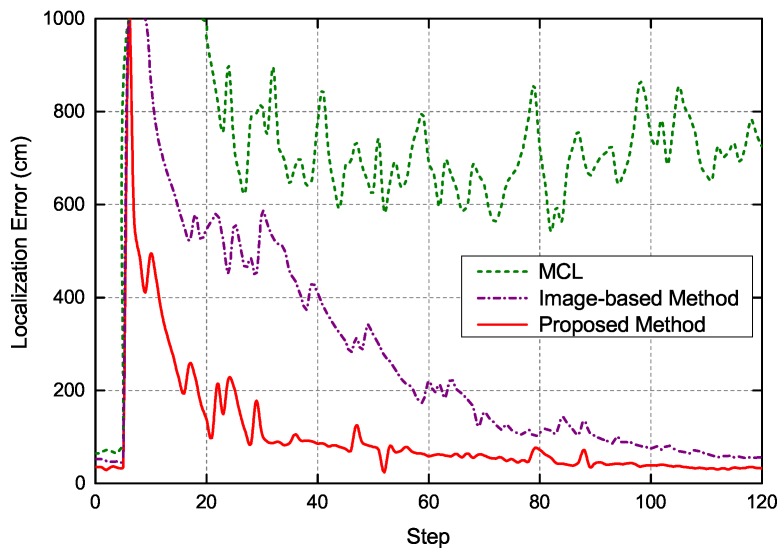
The motion path of the robot when it is kidnapped.

**Figure 12:**
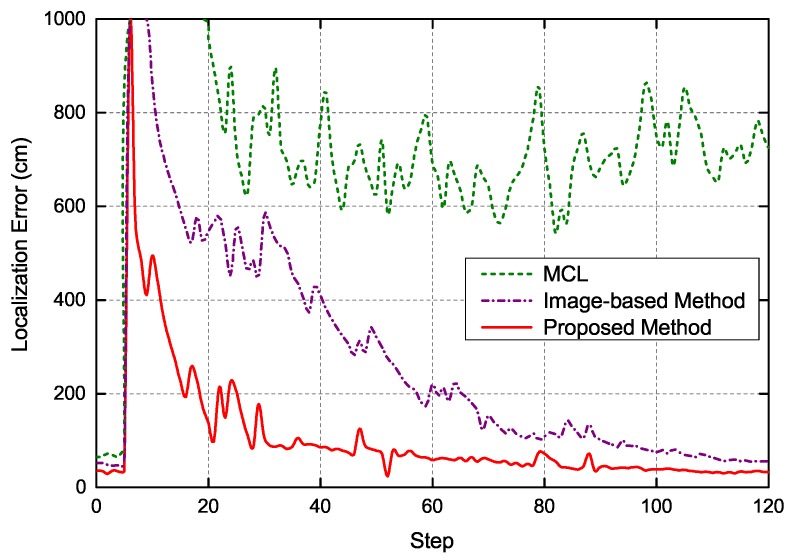
Typical localization error of three methods when the robot is kidnapped.

The parameters and procedure of this experiment were consistent with the global localization experiment presented above. As shown in Figure 11, the motion path of robot represented by blue lines started from Point A to Point B. When the robot moved to Point B, its pose was already determined. Then, the robot was kidnapped to Point C. In this case, the system needed to re-localize the robot. When kidnapped to Point C, the robot moved towards Point D. In the relocation stage, 1000 particles were randomly injected into the grid map at each execution step based on the results of image sequence processing with LSTM. Figure 12 presents the typical localization error of three localization methods when the robot was kidnapped. The robot was kidnapped after its accurate pose was already determined. As can be seen, the MCL could not effectively restore the location of robot. Although the image-based localization method enables the robot to escape from the kidnapping, it takes a long time to recover because its image-based re-localization is not accurate enough. In contrast, exploiting the correlation between image sequences, the proposed method could relocate the robot rapidly based on its powerful temporal sequence analysis capabilities. After the robot is kidnapped, the proposed method needs less than 40 steps to recover the location of robot and its localization error is less than 40 cm. Moreover, the robustness of proposed method is much higher than the other two localization methods. The experiment results demonstrate that the proposed localization method are indeed a promising avenue to tackle kidnapping problem in repetitive structures or similar layouts, which are predominant in modern indoor environment and are a tricky issue for traditional global localization methods.

## 5. Conclusions

In this work, we propose a novel multi-sensor-based indoor global localization system integrating visual localization aided by CNN-based image retrieval with a probabilistic approach denoted as Monte Carlo localization. The image retrieval performed with a pre-trained Faster RCNN model seeks to estimate the coarse locations of a mobile robot, which is taken as the input for the next stage of Monte Carlo localization. Besides, exploiting the correlation between image sequences, a closed-loop mechanism is introduced to deal with the robot kidnapping problem. When the robot is kidnapped, the LSTM network will be activated rapidly and make adaptive adjustments to provide a guidance for global localization. The integration of both techniques constructs a robust and efficient localization system. Systematic experiments were conducted on a real mobile robot. The results indicate that the proposed localization method exhibits great improvement on the speed and robustness of indoor localization compared to conventional localization methods. In addition, the proposed localization system enables the robot with the ability to recover from localization failure.

In the future, we plan to validate the localization system in different indoor environments such as low illumination and occlusion, which bring great disruption for the image retrieval. Moreover, we plan to further optimize the performance of image retrieval to suit various indoor environments.

## Figures and Tables

**Table 1:** Results of pose estimation relative to the query image from node 1.

	*X* (cm)	*Y* (cm)	θ (${}^{\circ }$)
Candidate node 1	1592.87	3720.63	−172.11
Candidate node 4	3681.13	304.76	94.25
Candidate node 5	−1548.34	3728.05	−169.68
Fine pose	1572.23	3723.51	−179.64
Ground truth	1561.45	3730.72	−173.42
Error	−10.78	7.21	6.22
